# Low Concentrations of Bisphenol A Induce Mouse Spermatogonial Cell Proliferation by G Protein–Coupled Receptor 30 and Estrogen Receptor-α

**DOI:** 10.1289/ehp.1103781

**Published:** 2011-08-03

**Authors:** Zhi-Guo Sheng, Ben-Zhan Zhu

**Affiliations:** 1 State Key Laboratory of Environmental Chemistry and Ecotoxicology, Research Center for Eco-Environmental Science, Chinese Academy of Sciences, Beijing, People’s Republic of China; 2 Linus Pauling Institute, Oregon State University, Corvallis, Oregon, USA

**Keywords:** 17β-estradiol, bisphenol A, estrogen receptor-α, G protein–coupled receptor 30, mouse spermatogonial GC-1 cells

## Abstract

Background: Bisphenol A (BPA) is one of the most prevalent chemicals in daily-use materials; therefore, human exposure to BPA is ubiquitous. The estrogenicity of BPA is generally mediated by nuclear estrogen receptors (ERs). However, low concentrations of BPA stimulate seminoma cell proliferation by an uncertain mechanism that does not involve activation of ERs.

Objective: We investigated the possible promoting effects of low-concentration BPA and the possible mechanism(s) using the murine ER-β negative spermatogonial GC-1 cell line.

Methods and results: Using the specific signaling inhibitor, BPA at test concentrations ranging from 10^–10^ to 10^–8^ M markedly induced proliferation of GC-1 cells by activating both cGMP-dependent protein kinase (PKG) and epidermal growth factor receptor (EGFR) extracellular regulated kinase (ERK) pathways. BPA stimulated a rapid (15-min) phosphorylation of the transcription factor cAMP response element binding protein (CREB) and the cell cycle regulator retinoblastoma protein (Rb). Interestingly, ER-α phosphorylation is involved in the proliferation, whereas BPA does not directly transactivate ER-α in gene reporter assays. Using specific agonists and gene silencing, we further observed that BPA mediates the proliferation and *fos* gene expression of GC-1 cells by G protein–coupled receptor 30 (GPR30) and ER-α.

Conclusions: Our data suggest that low concentrations of BPA activate the PKG and EGFR/ERK/c*-fos* pathways through a cross-talk between GPR30 and ER-α, which in turn stimulates GC-1 cell proliferation. The present study provides a novel insight regarding the potential role of GPR30 and ER-α in mediating the proliferative effects of BPA in male germ cells.

Bisphenol A (BPA) has been widely used in the manufacture of various consumer products and is one of the highest-volume chemicals produced worldwide. Hydrolysis of BPA under heat, acidic, or basic conditions leads to the release of BPA to the environment and the potential exposure to human beings. Indeed, available data from biomonitoring studies clearly indicate that the general population is exposed to BPA and is at risk from internal exposure to unconjugated BPA (Vandenberg et al. 2010). The core structure of BPA resembles that of natural 17β-estradiol (E2), and rodent and *in vitro* studies have demonstrated its endocrine-disruptive effects on reproductive and developmental tissues (Richter et al. 2007; Wetherill et al. 2007). However, there is an ongoing debate about whether BPA poses a hazard to human health at environmental exposure levels, because many xenoestrogens have weak binding ability to the classical estrogen receptors (ERs) 1,000–2,000 times lower than that of E2 at the lower picomolar or nanomolar concentrations (Bonefeld-Jørgensen et al. 2001). Recently, accumulating evidence has demonstrated that some xenoestrogens exert biological effects at levels lower than those presently considered safe. *In vivo* studies have documented that prenatal and neonatal exposure of male rats to low doses of BPA causes significant impairments in testicular development and spermatogenesis (Salian et al. 2011), suggesting a long-term and lasting impact of low-dose BPA on the offspring fertility during adulthood. Furthermore, BPA at environmentally relevant concentrations has been found to stimulate proliferation of human testicular JKT-1 cells through a nongenomic action independent of classical ERs (Bouskine et al. 2009). However, the exact mechanisms are still unclear.

In the present study, we used mouse spermatogonial GC-1 cells as an experimental model because these cells display specific features common to type B spermatogonia and early spermatocytes (Bellvé et al. 1977). In addition, GC-1 cells express G protein–coupled receptor 30 (GPR30), which mediates a rapid xenoestrogen-activated pathway in premeiotic germ cells, among which spermatogonia represent the most important cell population. Thus, these cell lines might be useful to investigate the role of GPR30 in the proliferation-promoting effects stimulated by BPA.

In the present study, we examined whether low concentrations of BPA (10^–12^ to 10^–7^ M), which are environmentally relevant, can stimulate proliferation of GC-1 cells via such a nongenomic action and, if so, whether GPR30 or its combination with ER-α is involved in the proliferation.

## Materials and Methods

*Reagents.* All chemicals were of reagent grade or better and were purchased from Sigma Chemical Co. (St. Louis, MO, USA) unless otherwise noted. We purchased antibodies against GPR30, ER-α, and glyceraldehyde 3-phosphate dehydrogenase (GAPDH) from Cell Signaling Technology Inc. (Beverly, MA, USA) and horseradish peroxidase-conjugated secondary antibody from Santa Cruz Biotechnology (Santa Cruz, CA, USA). All compounds were solubilized in dimethyl sulfoxide (DMSO) except E2 and PD98059 [2-(2-amino-3-methoxyphenyl)-4H-1-benzopyran-4-one], which were dissolved in ethanol. Although the quality of compounds was guaranteed by manufacturers, we confirmed quality by analyzing the key regents and solutions, such as BPA, before the experiments. We used steroid-free medium containing DMSO as the control.

*Cell treatments.* GC-1 and SkBr3 cells were cultured in phenol red–free Dulbecco’s modified Eagle medium (DMEM)/F-12 (1:1) and RPMI 1640 growth medium, respectively, which was supplemented with 10% charcoal-stripped fetal bovine serum, 1% glutamine, and 1% penicillin/streptomycin. Cells were maintained in growth medium for 48 hr and then switched to serum-free medium the day before immunoblot and reverse-transcription polymerase chain reaction (RT-PCR) experiments. For cell proliferation experiments, we used a cell density of 10^3^ cells per well as a stating point. Both the plastic items used for the experiments and the water used to prepare the reagents were pretreated by enhanced sonochemical degradation to reduce any potential background BPA (Pétrier et al. 2010).

*MTT [3-(4,5-dimethyl-thiazol-2-yl)-2,5-diphenyl-tetrazolium bromide] assay.* Cell viability was determined using an MTT assay. Briefly, at the end of treatment, 100 µL MTT (5 mg/mL in 1 M phosphate-buffered saline, pH 7.6) was added to each well of 96-well plates and the plates were incubated for 2 hr at 37°C in 5% CO_2_/95% air. Optical density (OD) was measured at 570 nm with a microplate reader.

*[^3^H]-thymidine incorporation analysis.* We evaluated [^3^H]-thymidine incorporation after 6 hr incubation with 1 µCi [^3^H]-thymidine per well. Cells were washed once with 10% trichloroacetic acid, twice with 5% trichloroacetic acid, then lysed in 1 mL 0.1 M NaOH at 37°C for 30 min. The total suspension was added to 10 mL optifluor fluid and counted in a scintillation counter.

*Caspase 3 activity assay.* We used the caspase 3 activity assay, which involves spectrophotometric detection of the chromophore *p*-nitroanilide after cleavage from the substrate Ac-DEVD-*p*-nitroanilide. The method has been described previously (Sheng et al. 2008).

*Effect of BPA on G protein–coupled receptor (GPCR), cGMP-dependent protein kinase (PKG), and epidermal growth factor receptor–extracellular regulated kinase (EFGR-ERK) pathways.* We examined BPA’s ability to activate different downstream signaling transduction pathways (kinases) by pretreating GC-1 cells with the PKG inhibitor KT5823, the ERK inhibitor PD98059, the Gαi/Gαq inhibitor pertussis toxin (PTX), the Src inhibitor PP2, the EGFR inhibitor AG-1478, the cAMP-dependent protein kinase (PKA) inhibitor H89, and the Gαs inhibitor NF449 before 12-hr exposure to 10^–9^ M BPA, a concentration that provided maximum cell proliferation and then evaluated cell viability using the MTT assay.

*Real-time RT-PCR.* Real-time RT-PCR methods have been described previously (Sirianni et al. 2008). Briefly, we obtained total RNA from cells using an RNA isolation kit (Invitrogen, Burlington, Ontario, Canada). After total RNA was spectrophotometrically quantified, we performed reverse transcription and PCR amplification using a One-Step Brilliant SYBR Green quantitative RT-PCR master mix kit (Bio-Rad, Hercules, CA, USA) in a single reaction following the manufacturer’s instructions. cDNA conversion, amplification, and data analysis were performed using an Mx3000P real-time PCR system computerized cycler (Bio-Rad). Primers for the amplification were based on published sequences for mouse *fos*. The nucleotide sequences of the primers were as follows: forward, 5´-GAG GAG GGA GCT GAC AGA TAC ACT-3´ and reverse, 5´-GAT TGG CAA TCT CAG TCT GCA A-3´.

*Western blot analysis.* Methods for Western blotting have been described in detail previously (Sheng et al. 2008). For protein extraction, treated cells were lysed in ice-cold lysis buffer, and the protein concentration was determined using a BCA (bicinchoninic acid) Protein Assay Kit (Invitrogen). Protein was separated using SDS-PAGE and then transferred onto a nitrocellulose membrane, which was subsequently probed with the following primary antibodies: anti-GPR30 (1:1,000), anti-ER-α (1:500), anti-*p*-Ser118-ER-α (1:500), anti-*p*-response element binding protein (CREB) (1:1,000), anti-*p*-retinoblastoma protein (Rb) (1:1,000), and anti-GAPDH as an internal control. After incubation, blots were incubated with the appropriate secondary antibodies conjugated to horseradish peroxidase. The bands were revealed by enhanced chemiluminescence using the ECL (enhanced chemiluminescence) commercial kit (Invitrogen).

*Plasmids.* Plasmids used were described previously by Albanito et al. (2008a). Briefly, we used EREtkLuc for ER and GK1 for yeast transcription factor Gal4 fusion proteins. EREtkLuc (XETL) contains the estrogen response element (ERE), the herpes simplex virus thymidine kinase promoter region, the firefly luciferase coding sequence, and the SV40 splice and polyadenylation sites. Gal4 chimeras Gal-ER-α and Gal-ER-β were expressed from plasmids GAL 93ER (G) and GAL ER-β, respectively. They were constructed by transferring the coding sequences for the hormone-binding domain (HBD) of ER-α from HEG0 and for the ER-β HBD from plasmid pCMV5-Her-β into the pSCTEVGal93. The *Renilla* luciferase expression vector pRL-TK served as a transfection standard.

*Transfection and luciferase assays.* We performed transfection and luciferase assays as described by Albanito et al. (2008a). Briefly, medium was replaced with phenol red–free DMEM/F-12 or RPMI 1640, and transfection was performed using FuGENE 6 Reagent (Roche Diagnostics, Mannheim, Germany) as recommended by the manufacturer. After the cells were subjected to the various treatment regimens, luciferase activity was measured with the Dual Luciferase Kit (Promega, Madison, WI, USA) according to the manufacturer’s recommendations. Firefly luciferase values were normalized to the internal transfection control provided by *Renilla* luciferase activity. The normalized relative light unit values obtained from cells treated with vehicle were considered 1-fold induction, and the activity induced by the various treatments was then calculated based on the control reference.

*Antisense oligodeoxynucleotide experiments.* We purchased antisense (AS) oligonucleotides (AS-ODNs) from MWG (Florence, Italy). The ODNs used were 5´-TGG AGT AGT CGC ATC CAT-3´ for *Gpr30*; 5´-GACCATGACCATGACCCT-3´ for *Ers-1*, and 5´-GATCTCAGCACGGCAAAT-3´ for the scrambled control. For AS experiments, 200 nM of the indicated ODN was transfected for 5–6 hr before treatment using Lipofectamine LTX (1:3; Invitrogen) according to manufacturer’s instructions. Cells were maintained in medium containing the transfection mix for 36 hr before being treated for 5 min and then lysed and used for Western blot analysis. For proliferation experiments, cells were maintained in medium containing the transfection mix for 24 hr, and medium was then replaced for the treatment.

*Statistical analysis.* All data were expressed as mean ± SE. We used the Student’s *t*-test for comparison of two groups in all the experiments. We considered differences with *p*-values < 0.05 at both tails to be statistically significant. All statistical analyses were performed using the SPSS software for Windows, Release 11.5 (SPSS, Chicago, IL, USA).

## Results

**Figure 1:**
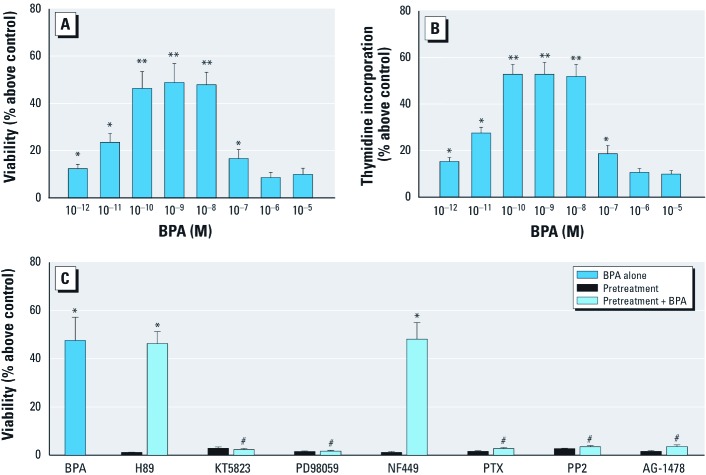
Twelve-hour exposure to low concentrations of BPA stimulated proliferation of GC-1 cells through activation of the GPCR, PKG, and EFGR-ERK pathways, as determined by the MTT assay (*A*) and by [^3^H]-thymidine incorporation (*B*), as described in “Materials and Methods.” (*C*) Viability of cells exposed to 10^–9^ M BPA for 12 hr with or without pretreatment with H89, KT5823, PD98059, NF449, PTX, PP2, or AG1478 (inhibitors of PKA, PKG, MAPK, Gαs, Gαi/ Gαq, Src, and EFGR, respectively). Values shown for *A–C* are the percentage change above the control (mean ± SE), with the control set as 1; results represent three independent experiments performed in triplicate. *p < 0.05, compared with control. **p < 0.05, compared with 10^–12^, 10^–11^, 10^–7^, 10^–6^, or 10^–5^ M BPA. ^#^*p* < 0.05, compared with 10^–9^ M BPA alone.

*Very low concentrations of BPA stimulate GC-1 cell proliferation.* GC-1 cells were exposed to BPA (10^–12^ to 10^–5^ M) for 12 hr, and cell proliferation was examined by MTT assay. BPA induced poliferation in an inverse U-shaped concentration-independent manner, with a weak promotion at 10^–7^ M and 10^–11^ M (23.56% and 16.64%, respectively) and the maximal effect around 10^–10^ to 10^–8^ M (46.28%, 48.81%, and 47.82%, respectively) [Figure 1A; see also Supplemental Material, Figure 1 (http://dx.doi.org/doi:10.1289/ehp.1103781)]. These results demonstrate that GC-1 cell proliferation is stimulated by BPA at very low concentrations. However, another interpretation of this proliferative effect is that BPA suppressed apoptotic regulating genes or promoted their antiapoptotic counterparts. However, we ruled out this possibility because BPA did not inhibit caspase 3, the final effector of apoptosis relative to controls (see Supplemental Material, Figure 2). Thus, BPA presumably does not enhance cell survival by obstructing normal apoptotic mechanisms.

**Figure 2:**
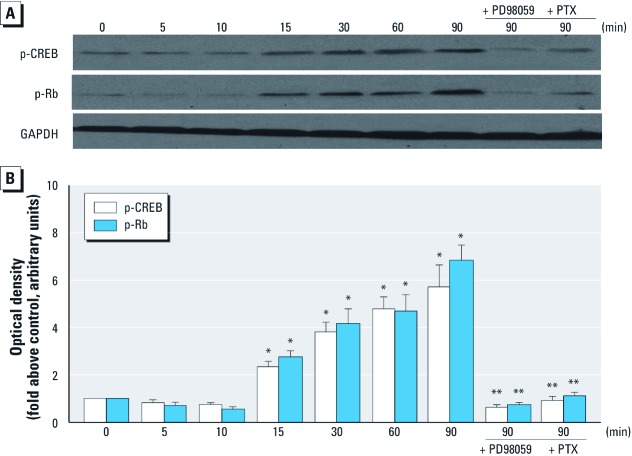
BPA rapidly induced protein expression of both phosphorylated CREB (p-CREB) and phosphorylated RB (p-Rb) in GC-1 cells by activating the PKG and ERK pathways. (*A*) Western blot analysis of cells treated with 10^–9^ M BPA for 5–90 min with or without pretreatment with PD98059 or PTX; GAPDH was used as internal control to ensure that equal amounts of protein were loaded in each lane. Data represent similar results from three independent experiments. (*B*) Bands from three experiments quantified by densitometry, with results (mean ± SE) normalized to GAPDH expression in each sample. *p < 0.05, compared with control (time 0). **p < 0.05, compared with time 90 min.

In addition, a [^3^H]-thymidine incorporation assay performed to corroborate data obtained with the MTT assay yielded results comparable with the viability assay [Figure 1B; see also Supplemental Material, Figure 3 (http://dx.doi.org/doi:10.1289/ehp.1103781)], thereby supporting the notion that very low concentrations of BPA significantly stimulate proliferation of GC-1 cells.

**Figure 3:**
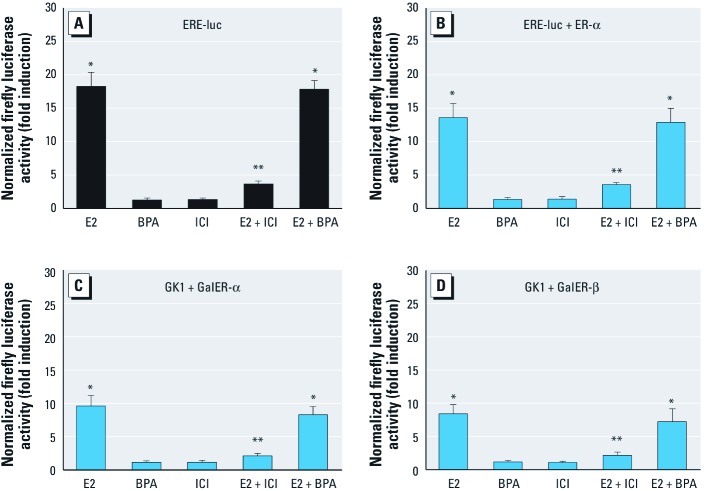
BPA did not directly transactivate ER-α in GC-1 and SkBr3 cells, as shown by firefly luciferase activity in GC-1 cells (*A*) and SkBr3 cells (*B*–*D*). (*A*) GC-1 cells transfected with the ER luciferase reporter plasmid ERE-luc (XETL) and treated with E2 or BPA (each at 10^–9^ M), with or without ICI. (*B*–*D*) SkBr3 cells transfected with ER luciferase reporter gene XETL and ER‑α expression plasmid (*B*), with Gal4 reporter gene (*GK1*) and the Gal4 fusion proteins encoding the HBD of ER (GalER‑α; *C*), and with *GK1* and GalER‑β (*D*) and treated with E2 or BPA (each at 10^–9^ M), with and without ICI. See “Materials and Methods” for details*.* Luciferase activity was normalized to *Renilla*luciferase expression vector (pRL-TK), and the value for vehicle-treated cells was set as 1-fold induction. Values shown (mean ± SE) represent the results of three independent experiments performed in triplicate. *p < 0.05, compared with control. **p < 0.05, compared with E2.

*BPA promotes GC-1 cell proliferation through activating both GPCR, PKG, and EFGR-ERK pathways.* Through activating different downstream signaling transduction pathways (kinases), xenoestrogens fine-tune cell proliferation or apoptosis (Bulayeva and Watson 2004). As shown in Figure 1C, the PKG inhibitor KT5823, the ERK inhibitor PD98059, the Gαi/Gαq inhibitor pertussis toxin (PTX), the Src inhibitor PP2, and the EGFR inhibitor AG-1478 prevented GC-1 proliferation by 10^–9^ M BPA, a concentration that provided maximum cell proliferation, whereas the PKA inhibitor H89 and the Gαs inhibitor NF449 did not, suggesting that both GPCR, PKG, and EGFR-ERK, but not PKA signaling pathways, were involved in proliferation stimulation by BPA.

*BPA rapidly stimulates the phosphorylation of both transcription factor CREB and nuclear factor Rb in GC-1 cells.* An earlier study revealed that the transcription factor CREB and the nuclear factor Rb participate in the regulation of gene transcription (Mayr and Montminy 2001). This regulation is related to the stage of the cell cycle and the phosphorylation induced by a variety of protein kinases, including PKG and ERK (Coulon et al. 2010; Mayr and Montminy 2001; Shaywitz and Greenberg 1999). Consequently, we wanted to determine if the well-recognized stimulus-induced transcription factor CREB and the cell cycle regulator Rb can be rapidly activated by BPA in a nongenomic manner. As shown in Figure 2, BPA exposure led to rapid (15 min) phosphorylation (activation) of CREB and Rb in GC-1 cells, which was dependent on ERK and PKG, as both PD98059 and PTX completely abolished this CREB and Rb phosphorylation.

*BPA-induced proliferation of GC-1 cells is involved in activation of classical nuclear ER-*α. BPA-stimulated proliferation was similar to that caused by E2-bovine serum albumin (BSA), an impermeable E2 conjugate [see Supplemental Material, Figure 4 (http://dx.doi.org/doi:10.1289/ehp.1103781)]. However, E2-BSA plus BPA induced an effect equivalent to those of E2-BSA of BPA alone, indicating a lack of synergistic or antagonistic effect and likely a convergence in the activated pathways. It should be noted that the combination of BPA and E2 significantly promoted the proliferative effects of E2, suggesting that ER-α may play a role in the stimulation in GC-1 cells by by BPA (see Supplemental Material, Figure 4). Predictably, the ER antagonist ICI significantly counteracted the proliferative effects of E2 and BPA, but not E2-BSA, on GC-1 cells, indicating a classical nuclear ER-α–dependent mechanism, because GC-1 cells express ER-α but not ER-β. Additionally, ER-α has an apparent intracytoplasmic localization without any evident membrane location (Sirianni et al. 2008). The above results demonstrated that the proliferative effects by BPA in GC-1 cells were likely dependent on both the classical ER-α and the GPCR-EFGR-ERK pathways.

**Figure 4:**
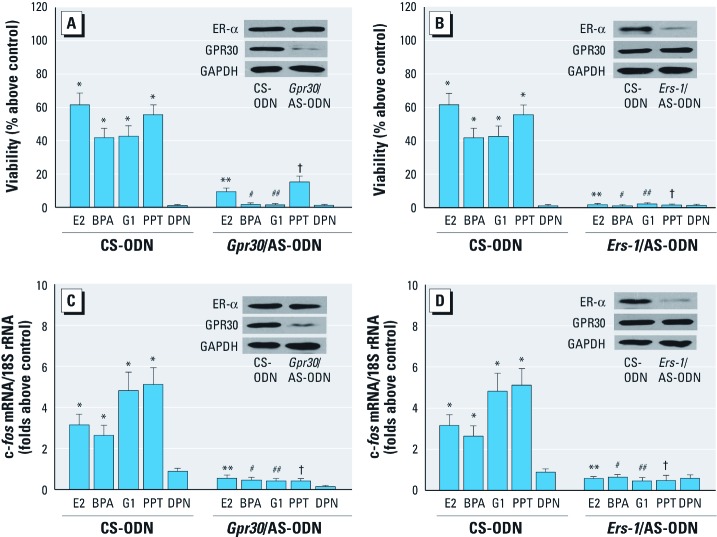
Silencing of *Gpr30* or *Ers-1* inhibited BPA-induced cell proliferation (*A, B*) and *fos* gene expression (*C, D*) in GC-1 cells transfected with control scrambled (CS)-ODN and GPR30/AS-ODN (*A, C*) or with CS-ODN and *Ers‑1*/AS-ODN (*B, D*) and treated with E2 (10^–9^ M), BPA (10^–9^ M), G1, PPT, or DPN (each at 10^–7^ M). Cell proliferation was determined by MTT assay (*A*,* B*), and *fos* mRNA levels were examined with real-time RT-PCR (*C*,* D*), as described in “Materials and Methods.” Values shown (mean ± SE) represent results of three independent experiments performed in triplicate; the control value was set as 1. *p < 0.05, compared with control. **p < 0.05, compared with E2. ^#^*p* < 0.05, compared with BPA. ^##^*p* < 0.05, compared with G1. ^†^*p* < 0.05, compared with PPT.

*BPA does not directly activate ER-*α *in GC-1 cells.* Based on the finding that ER-α was involved with BPA-induced proliferation in GC-1 cells, we further employed transfection assays to assess whether BPA directly activated ER-α. As shown in Figure 3A, exposure of the GC-1 cells to 10^–9^ M E2 strongly stimulated ER-α transactivation through a transiently transfected ER reporter gene but was abolished in the presence of 10 µM ICI. In contrast, BPA at ≥ 10^–9^ M (data not shown) failed to induce luciferase expression or to block that observed on addition of E2. Moreover, BPA did not activate an expression vector encoding ER-α transiently transfected in ER-negative SkBr3 breast cancer cell (Figure 3B). In another heterologous system, chimeric proteins consisting of the DNA binding domain of the yeast transcription factor Gal4 and the ER-α or ER-β HBD transiently transfected in SkBr3 cells were strongly activated by E2 but not by BPA (Figure 3C,D), further corroborating the aforementioned results. These data suggested that BPA failed to directly activate ER-α in GC-1 cells (ER-β negative); this result has also been reported in JKT-1 cells (Bouskine et al. 2009).

*BPA-stimulated proliferation of GC-1 cells is involved in activation of the GRP30.* Recently, Sirianni et al. (2008) showed that GPR30, an orphan GPCR, mediates the proliferative effects induced by E2 in GC-1 cells. Therefore, we examined whether both GPR30 and ER-α were involved in BPA’s promotion of effects in GC-1 cells. We observed that the specific GPR30 agonist G1 and the ER-α agonist propyl pyrazole triol (PPT), but not the ER-β agonist DPN, stimulated GC-1 cell proliferation [see Supplemental Material, Figure 5 (http://dx.doi.org/doi:10.1289/ehp.1103781)]. Furthermore, we found that BPA plus G1 also induced the same effect as each of them alone, as was the case with BPA plus E2-BSA, indicating a similarity in the activated pathways. However, the combination of PPT and BPA or PPT and G1 exhibited even greater proliferative effects than did PPT, BPA, and G1 alone (PPT plus BPA, *p* = 0.024; PPT plus G1, *p* = 0.019), suggesting that an additive or synergistic effect was possibly involved. These findings indicate that both GPR30 and ER-α were involved in proliferation of GC-1 cells, which was also confirmed by Sirianni et al. (2008), and that cross-talk likely exists between these two proteins.

*BPA phosphorylates the Ser118 site of ER-*α *by activating the GPR30 and EFGR-ERK pathways.* Mitogen-activated protein kinase (MAPK)-mediated phosphorylation in Ser118 site of ER-α has been suggested to enhance the tumor growth induced by MAPK/ER cross-talk (Atanaskova et al. 2002). We wanted to know if BPA could phosphorylate the Ser118 site of ER-α via such a mechanism. As shown in Supplemental Material, Figure 6 (http://dx.doi.org/doi:10.1289/ehp.1103781), 10^–9^ M BPA markedly increased the levels of *p*-ser118-ER-α (lane 2). However, the effects were completely abolished by the GPR30 antagonist G15 (lane 4), EFGR inhibitor AG-1478 (lane 6), or MAPK inhibitor PD98059 (lane 8). These results demonstrate that BPA activates ER by phosphorylating ER-α, and this response is likely mediated through the GPR30 and EFGR-ERK pathways.

*Silencing of* Gpr30 *and* Ers-1 *blocks BPA-induced proliferation and* fos *expression in GC-1 cells.* To further confirm the role that GPR30 and ER-α exert in BPA-induced proliferative effects of GC-1 cells, we investigated cell proliferation by reducing *Gpr30* and *Ers-1* expression by using AS-ODNs directed against these genes. The silencing of *Gpr30* and *Ers-1* expression by specific AS-ODNs significantly abolished GC-1 cell proliferation induced by E2, BPA, G1, and PPT without altering either *Ers-1* expression or *Gpr30* (Figure 4A,B). Predictably, the antisense *Gpr30* ODN could not completely suppress the proliferative effects of E2 and PPT, further suggesting that cross-talk between GPR30 and ER-α was involved with BPA-induced proliferation of GC-1 cells.

GPR30 mediates rapid estrogen signaling, leading to the induction of the activator protein-1 (AP-1) family member c*-fos* (Sirianni et al. 2008), which participates in the regulation of the cell cycle (Shaulian and Karin 2002). As shown in Figure 4C and 4D, the AS-ODN completely blocked the expression of *fos* mRNA induced by E2, BPA, G1, and PPT without altering *Ers-1* or *Gpr30* expression.

The induction of c*-fos* via GPR30 was previously demonstrated to occur after activation of EGFR and MAPKs (Sirianni et al. 2008). As we predicted, both the EGFR inhibitor AG-1478 and the ERK inhibitor PD98059 significantly inhibited *fos* expression induced by E2, BPA, G1, and PPT [see Supplemental Material, Figure 7 (http://dx.doi.org/doi:10.1289/ehp.1103781)]. In addition, we also observed that ICI markedly decreased BPA-mediated *fos* levels (see Supplemental Material, Figure 7), suggesting that ER-α participated in the induction of BPA to *fos.*

## Discussion

In this study we demonstrated that low concentrations of BPA (10^–10^ to 10^–8^ M) stimulated mouse spermatogonial proliferation of GC-1 cells by activating both PKG and EFGR-ERK-c-*fos* pathways mediated via cross-talk between GPR30 and ER-α.

Low concentrations of BPA have been reported to trigger a nongenomic proliferative effect in the pancreatic islet, endothelium, breast, and pituitary gland by initiating rapid responses (Bulayeva and Watson 2004; Wetherill et al. 2007). In the present study, we also observed a representative nongenomic proliferative effect, which presented as the paradoxical inverse U-shaped curve, and rapid activation of CREB and Rb. Bouskine et al. (2009) demonstrated that low concentrations of BPA promote proliferation in JKT-1 human seminoma cells by activating PKA and PKG via a membrane GPCR. However, in our study, membrane GPCR, EFGR-ERK, and PKG pathways participated in the proliferation of GC-1 cells stimulated by BPA. Transactivation of the EGFR and the ERK/MAPK cascade promoted by the GPCR agonist has been shown in a variety of cellular contexts. Again, estrogenic activation of the ERK/CREB pathway via a GPCR has already been demonstrated in several models (Belcher et al. 2005; Filardo 2002).

We observed that ER-α was involved in BPA’s activity because gene silencing and antagonism of ER-α significantly suppressed BPA-stimulated proliferation of GC-1 cells. However, the activation of ER-α was not through its direct binding with BPA in the gene reporter assay, but it was phosphorylated at its Ser118 site. ER-α contains both hormone-independent and hormone-inducible transactivation functions (AF-1 and AF-2, respectively) (Metzger et al. 1995). Hormone-independent activation of ER-α by growth factors leads to phosphorylation of Ser118 located within AF-1 (Joel et al. 1995). Because many growth factor pathways converge on MAPK, it is thought that MAPK-mediated phosphorylation of AF-1 represents a mechanism whereby MAPK/ER cross-talk enhances ER-α–mediated signaling and tumor growth (Atanaskova et al. 2002). Several lines of evidence have suggested that the interaction of EGFR-MAPK with estrogen signaling can occur at different levels (Filardo et al. 2000; Levin 2003). Indeed, our study reveals that BPA phosphorylates ER-α by EFGR-ERK–dependent pathways.

An unknown GPCR has been suggested to mediate the BPA-stimulated proliferation of GC-1 cells because of the suppression of the Gαi/Gαq inhibitor PTX and promotion of E2-BSA, an impermeable E2 conjugate. Recently, GPR30, a transmembrane estrogen-binding orphan GPCR, has been shown to potentially mediate rapid E2-dependent proliferation in cancer cells (Albanito et al. 2007; Filardo 2002; Revankar et al. 2005). A variety of environmental contaminants exhibit binding affinities for GPR30 as well as agonist activities similar to those for ER (Thomas and Dong 2006). The present data indicate that GPR30 is required for this proliferation-promoting response and for indirect activation of ER-α phosphorylation by BPA.

c-*fos* represents a prototypical early gene, and its encoded nuclear protein regulates the expression of genes involved in proliferation, invasion, differentiation, and cell survival (Shaulian and Karin 2002). The present study indicates that BPA induces *fos* gene expression by the activation of GPR30, EFGR-ERK, and ER-α. GPR30 expression is induced through an EGFR-MAPK-c*-fos* pathway by recruiting c*-fos* to the AP-1 site of the *GPR30* gene, aggravating GPR30 and c*-fos* expression via a regulatory loop (Albanito et al. 2008b). Thus, a significant increase (3- to 5-fold) in *fos* expression after the various treatments observed in the present study will assuredly bolster the BPA-induced proliferation of GC-1 cells, because the subtle change in *fos* gene expression equated to profound phenotypic effects.

Estrogens acting via GPR30 are capable of stimulating adenylyl cyclase activity, which in turn leads to PKA-mediated suppression of EGFR-ERK (Filardo et al. 2002). Our results suggest that PKA possibly did not participate in the suppression of this pathway because inhibition of PKA activation could not enhance the proliferative effects of BPA. Thus, we assumed that BPA-induced proliferation of GC-1 cells via GPR30 was likely to be long-lasting because of the lack of the opposing effects on the EGFR-to-MAPK axis.

Hence, data from the present study indicate that a complex interplay between ER-α and GPR30 contributes to low concentrations of BPA activity in GC-1 cells by activating EGFR-ERK transduction pathways, similar results for atrazine in ovarian cancer cells (Albanito et al. 2008a; Bunone et al. 1996). Based on the present data and available literature, BPA may act via GPR30 to activate PKG and induce the release of surface-bound, membrane-anchored, heparin-binding, EGF-like growth factor (proHB-EGF) (Filardo et al. 2000), which in turn activates EFGR-ERK/ER-α/c*-fos* pathways, finally leading to the stimulation of a mitogenic signaling network (Bunone et al. 1996; Lannigan 2003).

ER-β participates in the control of proliferation and/or apoptosis of male germ cells just after birth, and its down-regulation is strongly correlated with human testicular seminomas (Hirvonen-Santti et al. 2003; Pais et al. 2003). Therefore, it has been assumed that human gonocytes, which do not express the active ER-β1 isoform until the prenatal period (Gaskell et al. 2003), may be exclusively sensitive to the BPA-mediated promoting effect. Thus, the exposure of the fetuses to BPA likely poses a hazard to male germ cells. In a group of pregnant mothers and their fetuses, Schönfelder et al. (2002) observed blood BPA levels of 0.3–18.9 ng/mL (1.31 × 10^–9^ to 8.28 × 10^–8^ M) and 0.2–9.2 ng/mL (8.76 × 10^–10^ to 4.29 × 10^–8^ M), respectively; based on prior studies (Welshons et al. 2006), these levels equate to similar concentrations used in the present study. This implies that exposure of pregnant mothers to low BPA levels may exert adverse biological effects on fetuses. Thus, excessive fetal exposure to xenoestrogens with high affinity for the nonclassical estrogen GPCR (e.g., GPR30), such as shown here for BPA, may stimulate abnormal nongenomic proliferation of gonocytes, consequently resulting in malignant germ cell transformation/carcinoma *in situ* and then testicular germ cell cancer, the most frequent cancer of young men, with increasing incidence.

The primary focus of our study was on an *in vitro* approach to investigate the molecular mechanisms of BPA action. However, it is critically important to consider combining *in vitro* and *in vivo* approaches to investigate actions of BPA at exposures mirroring those that occur in human and animal populations. Thus, in future research, we will extend our *in vitro* experiments to an *in vivo* study using rats chronically exposed to environmentally relevant concentrations of BPA.

In conclusion, our results provide a novel insight regarding the potential role of GPR30 and ER-α in mediating a growth stimulatory action of low concentrations of BPA in male germ cells.

## Supplemental Material

(233 KB) PDF
