# Supplementary material for: Low Concentrations of Bisphenol A Induce Mouse Spermatogonial Cell Proliferation by G Protein–Coupled Receptor 30 and Estrogen Receptor-α
Source: Environ Health Perspect. 2011 Aug 3;119(12):1775–80. doi: 10.1289/ehp.1103781 (PMC3261991; doi:10.1289/ehp.1103781)
Supplement: (233 KB) PDF [file ehp.1103781.s001.pdf]

Supplemental Material

Low Concentrations of Bisphenol A Induce Mouse Spermatogonial Cell Proliferation by G Protein-Coupled Receptor 30 and Estrogen Receptor-α

Zhi-Guo Sheng[1] and Ben-Zhan Zhu[1,2]

[1]State Key Laboratory of Environmental Chemistry and Ecotoxicology, Research Center for Eco-Environmental Science, Chinese Academy of Sciences, Beijing 100085, P.R. China; [2]Linus Pauling Institute, Oregon State University, Corvallis, OR 97331

Table of Contents

**Supplemental Material, Figure 1**	**P. 3**
**Supplemental Material, Figure 2**	**P. 4**
**Supplemental Material, Figure 3**	**P. 5**
**Supplemental Material, Figure 4**	**P. 6**
**Supplemental Material, Figure 5**	**P. 7**
**Supplemental Material, Figure 6**	**P. 8-9**
**Supplemental Material, Figure 7**	**P. 10**

Supplemental Material, Figure 1

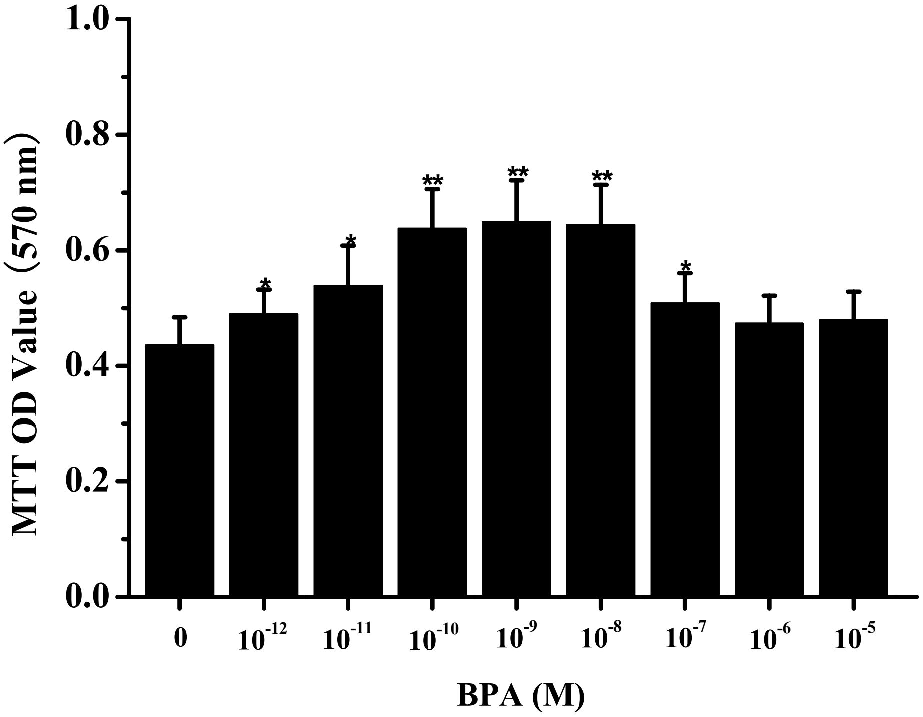

Proliferative effects of BPA on GC-1 cells. Cells were exposed to the indicated concentrations of BPA incubated for 12 h. cell proliferation was assessed by MTT assay, as described in *Materials and Methods*. The results of three independent experiments performed in triplicate were shown as mean ± SD. *, $p < 0.05$, compared with control; **, $p < 0.05$, compared with 10^{-12}, 10^{-11}, 10^{-7}, 10^{-6}, or 10^{-5} M BPA;

Supplemental Material, Figure 2

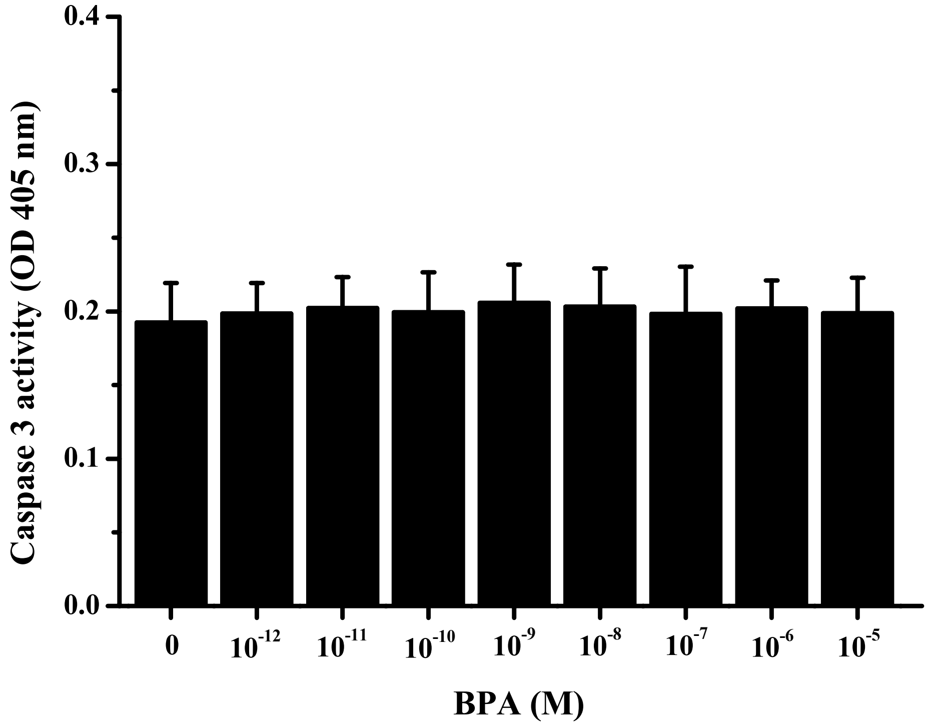

Low concentrations of BPA do not suppress the caspase 3 activity of GC-1 cells. The activity of caspase-3 was measured using the caspase-3-specific tetrapeptide substrate Ac-DEVD-*p*-nitroanilide, as described in *Materials and methods*. The values were means ± SEM of three independent experiments.

Supplemental Material, Figure 3

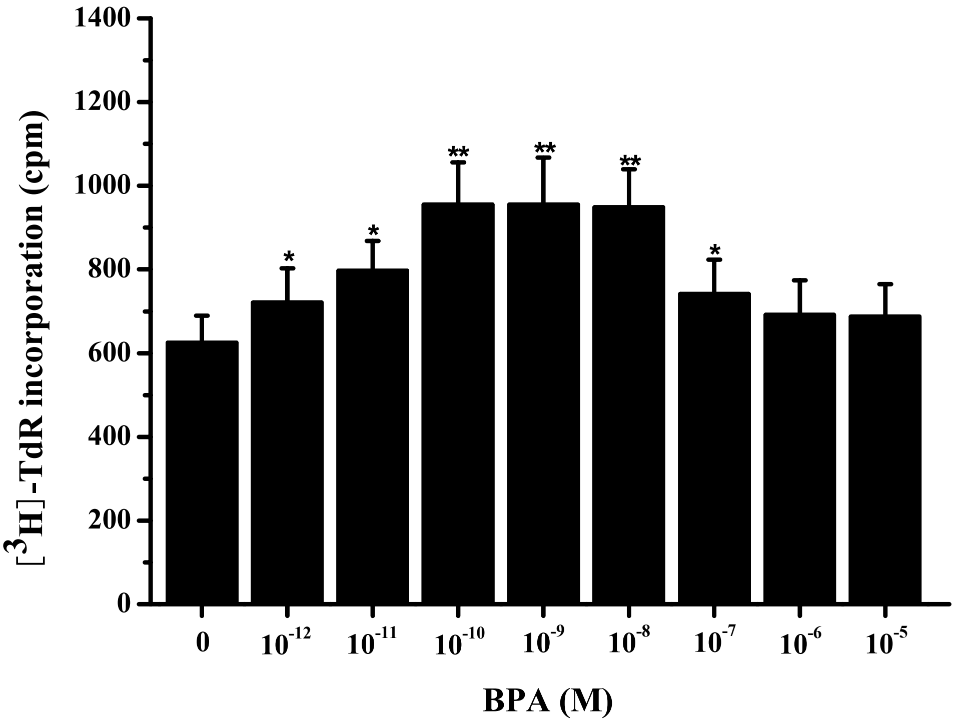

[^{3}H]-TdR incorporation effects of BPA on GC-1 cells. Cells were exposed to the indicated concentrations of BPA incubated for 12 h. cell proliferation was assessed by [^{3}H]-TdR incorporation analysis, as described in *Materials and Methods*. The results of three independent experiments performed in triplicate were shown as mean ± SD. *, $p < 0.05$, compared with control; **, $p < 0.05$, compared with 10^{-12}, 10^{-11}, 10^{-7}, 10^{-6}, or 10^{-5} M BPA;

Supplemental Material, Figure 4

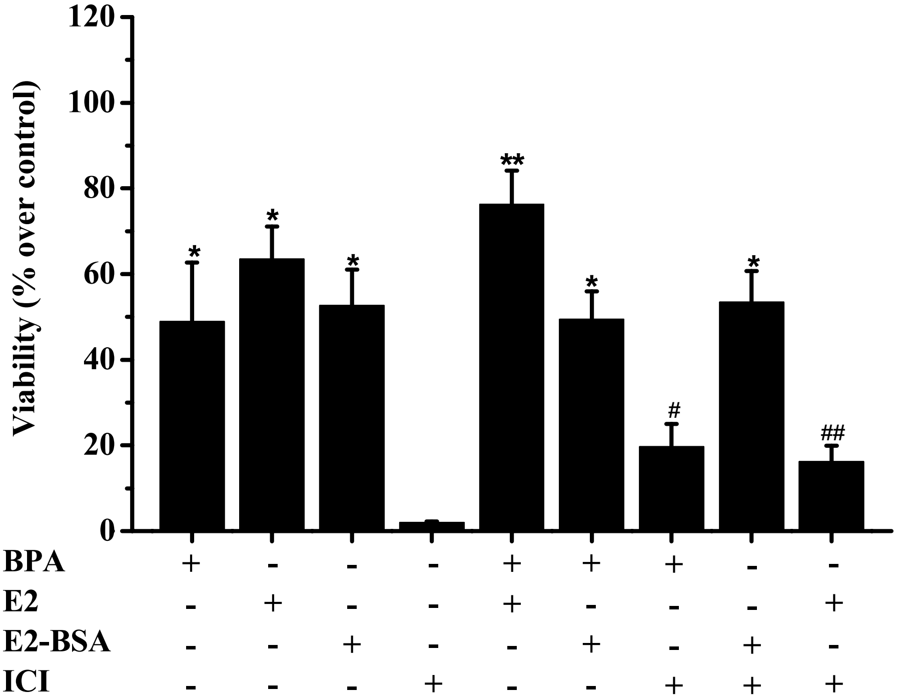

BPA-mediated GC-1 cell proliferation is likely through activating the membrane GPCR and the nuclear ER-α. Cells were exposed to 10^{-9} M BPA, combined with E2 or E2-BSA (each at 10^{-9} M) for 12 h or pretreatment with ICI for 60 min. Cell proliferation was determined by MTT assay, as described in *Materials and Methods*. The results of three independent experiments performed in triplicate were shown as mean ± SEM. The values shown were the percent change of viable cells compared with the control (steroid-free medium containing DMSO for BSA or ethanol for E2 and E2-BSA) and control was set as 1. *, $p < 0.05$, compared with control; **, $p < 0.05$, compared with E2; $^{\#}$, $p < 0.05$, compared with BPA alone; $^{\#\#}$, $p < 0.05$, compared with E2.

Supplemental Material, Figure 5

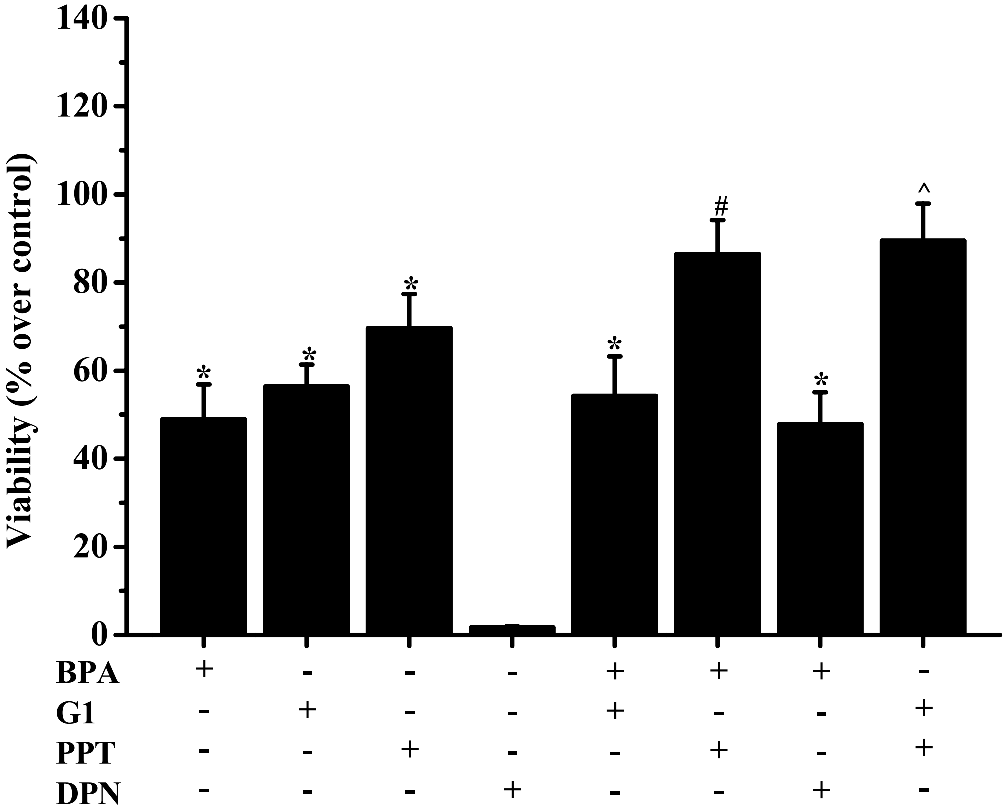

GPR30 and ER agonists stimulate GC-1 cell proliferation. Cells were treated with BPA (10^{-9} M), G1, PPT or DPN (each at 10^{-7} M) alone or combination. Cell proliferation was determined by MTT assay, as described in *Materials and Methods*. The results of three independent experiments performed in triplicate were shown as mean ± SEM. The values shown were the percent change of viable cells compared with the control (steroid-free medium containing DMSO) and control was set as 1. *, $p < 0.05$, compared with control; #, $p < 0.05$, compared with BPA or PPT; ^, $p < 0.05$, compared with G1 or PPT.

Supplemental Material, Figure 6

A

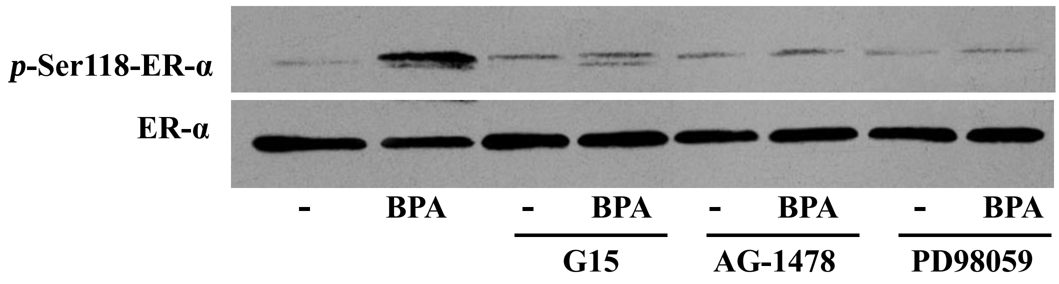

B

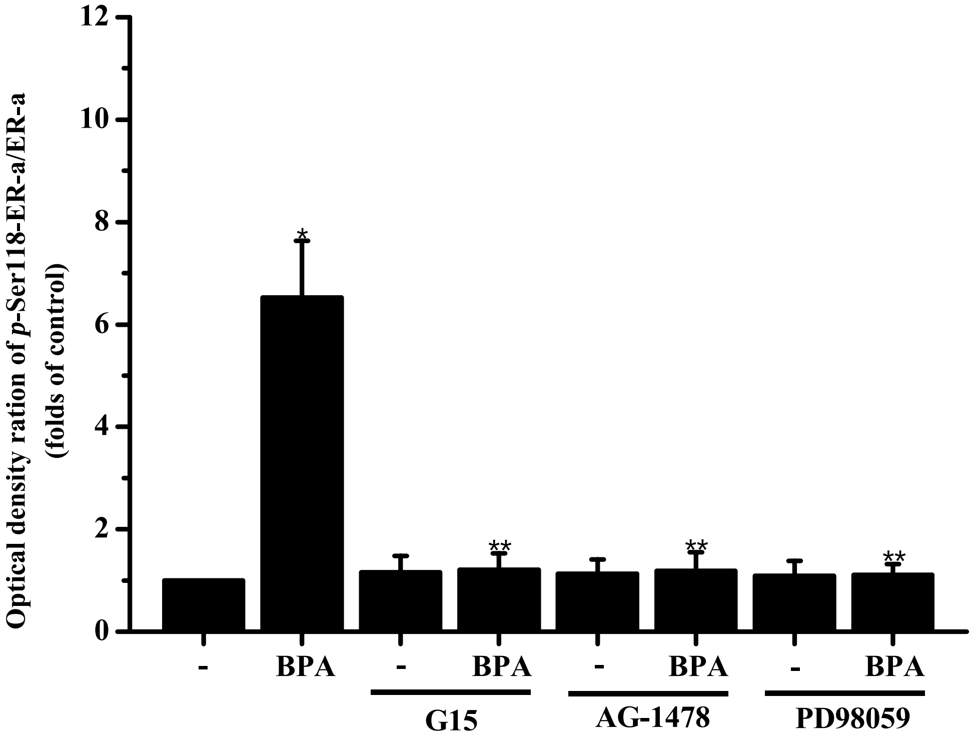

BPA activates the ER-α of GC-1 cell by GPR30-EFGR-ERK signaling pathway. (A) Cells were treated with 10^{-9} M BPA for 12 h combined with G15, AG-1478, or PD98059. Western blot analysis was used to examine both *p*-ser118-ER-α and total ER-α protein level, as described in *Materials and Methods*. The data represented similar results from three independent experiments performed in triplicate. (B) The

bands of *p*-Ser118-ER-α and ER-α were quantified by densitometry analysis, and the activated *p*-Ser118-ER-α was expressed as the *p*-Ser118-ER-α/ER-α ratio, plotted with SEM. *, $p < 0.05$, compared with control; **, $p < 0.05$, compared with BPA alone.

Supplemental Material, Figure 7

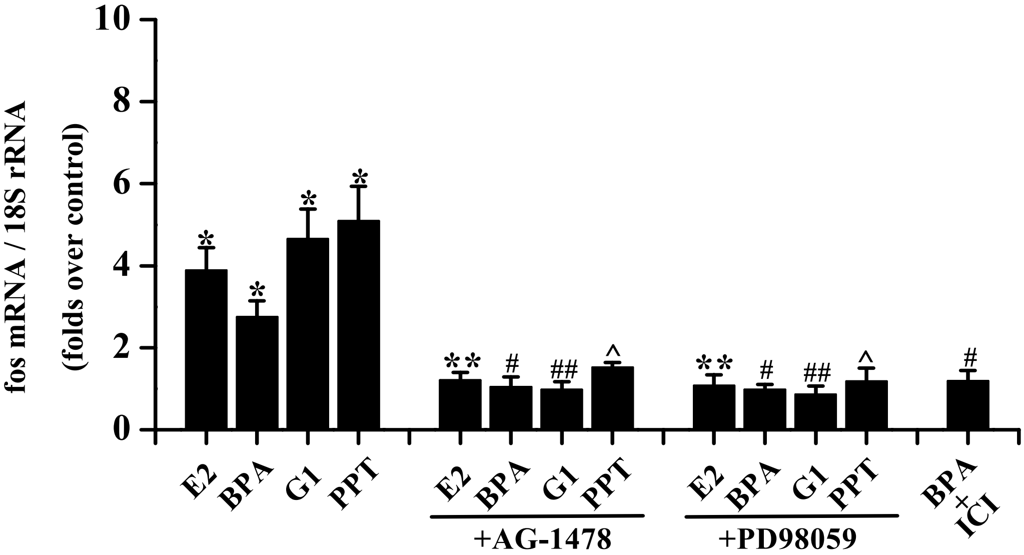

BPA stimulates the *fos* gene expression in GC-1 cells through activating the EFGR-ERK and ER-α pathways. Cells were treated with BPA (10^{-9} M), E2 (10^{-9} M), G1, PPT (each at 10^{-7} M) for 12 h combined with AG-1478 (10 μM), PD98059 (10 μM) or ICI (10 μM). Total RNA was extracted, and real-time RT-PCR was used to examine *fos* mRNA levels, as described in *Materials and Methods*. Each sample was normalized to its 18S rRNA content. The results of three independent experiments performed in triplicate were shown as mean ± SEM. *, $p < 0.05$, compared with control; **, $p < 0.05$, compared with E2 alone; $^{\#}$, $p < 0.05$, compared with BPA alone; $^{\#\#}$, $p < 0.05$, compared with G1 alone; ^, $p < 0.05$, compared with PPT alone.
